# Mixed method study of feasibility and acceptability of electronic screening for measurement-based symptom monitoring of veterans accessing mental health treatment in VA community care program settings

**DOI:** 10.1186/s12913-024-12029-1

**Published:** 2025-01-03

**Authors:** Erin Almklov, Michael W. Lee, John D. Gault, Brian H. Blanco, Brian Huynh, Abigail Angkaw, Neal Doran, Niloofar Afari, James O. E. Pittman

**Affiliations:** 1 VA San Diego Healthcare System, 3350 La Jolla Village Dr., San Diego, CA USA; 2 VA Center of Excellence for Stress and Mental Health, 3350 La Jolla Village Dr., San Diego, CA USA; 3 Department of Psychiatry, University of California San Diego, 9500 Gilman Drive, La Jolla, CA USA

**Keywords:** Veterans, Mental health, Community care, Measurement based care, eScreening

## Abstract

**Background:**

2022 survey data showed 29% of Veterans utilized Veterans Affairs (VA) paid health care at a non-VA facility, 6% higher than in 2021. Despite an increase in the number of Veterans accessing care in the community via the MISSION Act Community Care Program (CCP), there is limited information on the quality of mental health care delivered to Veterans in these settings. Further, Veterans report barriers to quality care, including poor communication between CCP and VA providers, which can result in negative patient outcomes. We aimed to evaluate the feasibility and acceptability of using electronic screening, eScreening, as part of a process involving remote symptom screening, symptom monitoring, and clinically driven communication from VA to CCP providers, for Veterans accessing mental health treatment in CCP settings.

**Methods:**

Veterans (*n* = 150) diagnosed with major depressive disorder, an anxiety disorder, post-traumatic stress disorder, and/or an adjustment disorder referred to mental health care in CCP between August-November 2021 were eligible to participate. Veterans received an eScreening link to complete an initial web-based assessment and three follow-up assessments spaced 4–6 weeks apart over the course of their treatment. Quantitative assessment data was largely characterized using descriptive statistics and included patient-reported outcome (PRO) measures (PTSD and depression), health-related quality of life/functioning, community care information (e.g., number of sessions attended), and satisfaction with the eScreening technology. Qualitative interview data was also collected from participating Veterans and CCP providers to better understand experiences with eScreening.

**Results:**

Findings support the feasibility and acceptability of using eScreening to administer and monitor PROs for Veterans accessing mental health treatment in CCP. Of the Veterans who provided eScreening satisfaction ratings (Ns = 45–55), 89% had no technical difficulties; 78% felt comfortable entering personal information; and 83% were neutral or positive about ease of use. Focus group interviews revealed strong support from Veterans, who stated the software was easy to use; they felt comfortable completing PRO measures; and they appreciated having their symptoms monitored. Similarly, providers indicated eScreening had a positive impact on communication, collaboration of care, and transparency.

**Conclusions:**

Technologies like eScreening represent a promising tool to support the mental health care Veterans receive when they access CCP.

## Background

Under the VA MISSION Act, the Department of Veterans Affairs (VA) provides care to eligible Veterans through community providers when VA Medical Centers cannot provide the care needed. The number of Veterans authorized to receive health care in non-VA settings, via the MISSION Act Community Care Program (CCP), has increased annually since 2014, with 33 million appointments completed in 2021 [1]. VA spending on community care has more than doubled from nearly 8 billion in 2014 to approximately 18.5 billion in 2021 [2]. The expansion of Veterans’ health care options has the potential advantage of giving many Veterans more freedom to decide which settings best meet their needs, but it has come with concerns and unintended challenges as well. Specifically, information on the quality of mental health care delivered to Veterans in CCP settings is scarce. Further, Veterans have identified barriers in relation to community mental health services, including awareness of available services, short appointments, provider continuity, providers’ lack of knowledge of military culture, stigma, and administrative challenges, such as poor communication between community providers and the VA [3, 4]. Poor communication can diminish the coordination and safety of Veterans’ care if critical information obtained in the community is not available or communicated to VA providers and vice versa [5, 6]. Systems are needed to facilitate the collection and communication of health information between VA and CCP clinicians to ensure that Veterans’ care is effective, well-coordinated, and safe [6].

Electronic self-report screening has been shown to be an effective assessment tool for timely detection and intervention of suicidal ideation and other mental health symptoms [7, 8]. Research also demonstrates that electronic self-report screening encourages patient-provider communication and aids in follow-up care [7]. The VA eScreening program is a secure web-based, patient-facing screening and information-provision system developed with Veteran and staff feedback that allows for rapid capture of self-report data and can improve care quality and coordination [9, 10]. It was created by the VA Center of Excellence for Stress and Mental Health with funding from the VA Innovations Ecosystem to facilitate early identification of Veteran needs in care coordination programs. Across 38 VA facilities, eScreening has been utilized over 100,000 times with Veterans in over 20 different clinical settings (e.g., Military2VA, Mental Health, Spinal Cord Injury, Whole Health, Caregiver Support, etc.). In 2016, the eScreening program was named a Gold Standard Practice for diffusion throughout VA by the Under-Secretary for Health [11] and it is now available nationwide.

eScreening provides actionable, real-time scoring and feedback, and integrates into the VA electronic medical record system. It supports a variety of patient self-report data elements and is unique among all other available systems in VA because it can easily be customized to any program- or provider-specific needs (e.g., display text, selection of measures/screens, Veteran feedback summary, automated medical record note text). eScreening has been used as a standard method for collecting patient reported outcomes (PRO) at the VA San Diego Healthcare System. PROs are standardized, validated survey tools that assess health outcomes (e.g., mental health symptoms) reported by patients [12]. They are used to track or report on the performance of healthcare providers and healthcare delivery organizations with an aim of improving the quality of healthcare services [12]. The regular assessment of specific PROs over time is highly important to inform treatment decisions, such as when to increase or decrease the level of care, alter treatment plans and interventions, or adjust treatment goals [13]. Routine use of PRO measures in clinical care is associated with benefits, including improvements in communication, quality management, and patient outcomes [14].

As the number of Veterans accessing care in the community (via the CCP) increases, so does the need to ensure effective, high quality, and coordinated care. Given the functionality, versatility, and increasing use nationally, eScreening could be a valuable tool to use with Veterans receiving mental health treatment in the community. Specifically, eScreening can be used by VA to remotely collect important PRO data, monitor outcomes over time, and signal when communication with a CCP provider is needed (e.g., if data indicate an important change in symptoms or functioning). Therefore, using a mixed methods design, this project aimed to evaluate the feasibility and acceptability of using eScreening as part of a remote VA directed process involving routine administration of PRO measures, outcome monitoring, and clinically driven communication from VA to CCP providers, for Veterans accessing mental health treatment in CCP settings.

## Methods

### Participants

All Veterans with a diagnosis of major depressive disorder, an anxiety disorder, post-traumatic stress disorder (PTSD), and/or an adjustment disorder referred to mental health care (i.e., individual psychotherapy) in the CCP between August 2021 through November 2021 were eligible to participate. Veterans were excluded if they were referred for other mental health conditions or were no longer receiving treatment in CCP due to consults being cancelled because of Veteran unresponsiveness, failure in scheduling efforts with CCP providers, or no longer being engaged in CCP.

### Procedures

Eligibility was determined via chart review conducted by a member of the project team. A VASDHS Mental Health Social Worker (MHSW) or Clinical Psychologist was notified of eligible participants and provided Veterans with an overview of the project by telephone, which include the following description: “As part of your participation in community care, VA San Diego Mental Health is going to send you a link to complete a web-based self-report health screening using eScreening. This will allow VA San Diego to collect information about your mental health symptoms and functioning as you receive treatment in the community so that we can ensure that you are getting the care you need. If there are concerns based on your responses, we will reach out to you to check in and assess to see if you need additional services. We will also let your community provider know so they can check in with you. Next, we will be sending you an email with a link to eScreening now and monthly throughout your course of treatment. It’s very important that these are completed consistently to ensure our best response to your needs while outside the VA. If you have any problems accessing eScreening, the email will list the support person for you to contact.” The CCP providers were also notified about the project. Thirty-six CCP providers were contacted by the study coordinator to inform them they had patient(s) participating in this project. The VA San Diego Institutional Review Board (IRB) waived the ethics approval and informed consent as this project was considered quality improvement in nature and determined by the IRB not to constitute research (reference ID: HRD210087).

Veterans who met inclusion were sent an eScreening link via email for an initial assessment, written instructions on how to access and complete the eScreening assessment and contact information for technical support. Following completion of the initial assessment, Veterans were sent an eScreening link via email to complete three follow-up assessments spaced approximately four to six weeks apart (Table 1). Nonrespondents received two reminders per week by email or telephone. A project team member monitored the eScreening dashboard daily for Veterans who completed assessments. Veteran assessments were automatically scored and pushed to the VA electronic medical record system as a note assigned to the MHSW. The MHSW monitored results of completed screens and followed up with Veterans and CCP providers as needed if there were clinical increases in symptoms, as defined as an endorsement of suicidal ideation on question 9 of the Patient Health Questionnaire-9 (PHQ-9) or a 5-point increase on the PHQ-9 or a 10-point increase on the PTSD Checklist for DSM-5 (PCL-5).

**Table 1:** Assessment schedule

Assessment	Measures
Initial	Demographic data; mental health measures (PHQ-9, PCL-5^a^, and VR-12)
Follow-up 1 (4–6 weeks after initial assessment)	Mental health measures (PHQ-9, PCL-5^a^, and VR-12); community care treatment information; eScreening survey
Follow-up 2 (4–6 weeks after follow-up 1)	Mental health measures (PHQ-9, PCL-5^a^, and VR-12); community care treatment information; eScreening survey
Final (4–6 weeks after follow-up 2)	Mental health measures (PHQ-9, PCL-5^a^, and VR-12); community care treatment information; eScreening survey

^a^Only included for participants with a PTSD diagnosis

Focus groups were conducted with two groups of the participating Veterans (*N* = 13). After a minimum of two months participation in regular screening, Veterans were invited to participate in a focus group interview to provide feedback about their experiences. Interview guides were developed to explore Veterans’ experiences with using eScreening and perceived impact on treatment. Qualitative focus group interviews were audio-recorded and transcribed verbatim by study staff*.* Using the same methods, the study team conducted individual interviews with seven CCP providers with a high volume of VA patients. The focus of these interviews was on willingness to use PRO data to inform their care with Veterans and on the perceived benefits and drawbacks of the care coordination model.

### Measures and data sources

*Demographics* include gender and race/ethnicity.

*The PCL-5* [15] is a 20-item self-report measure of PTSD symptoms corresponding to DSM-5 diagnostic criteria. Respondents rate how much they are bothered by PTSD symptoms over the past month on a scale from 0 to 4 (“not at all” to “extremely”). Total scores range from 0 to 80. Previous research has demonstrated that the PCL-5 has excellent psychometric properties, including strong convergent validity with the Impact of Event Scale–Revised (IES-R), a well-established measure of PTSD symptom severity, (r = 0.82, *p*< 0.001), and excellent internal consistency, α = 0.95 [16].

*The PHQ-9* [17] is a 9-item self-report measure of depressive symptoms using a 4-point rating scale ranging from 0 (“not at all”) to 3 (“nearly every day”). The maximum score is 27, with higher scores indicating greater severity of depressive symptoms. The PHQ-9 has strong psychometric properties, including good internal consistency, test–retest reliability, and convergent validity [17].

*The Veterans RAND Health Survey* VR-12 [18] is a 12-item self-report health survey used to measure health related quality of life. The Likert-type items correspond to different physical and mental health domains including general health perceptions; physical functioning; role limitations due to physical and emotional problems; bodily pain; energy-fatigue; social functioning and mental health. The 12 items are summarized into two scores, a Physical Health Summary Measure (PCS) and a Mental Health Summary Measure (MCS), with higher summed scores indicating better health. The VR-12 has been shown to be a reliable and valid PRO measure that is also responsive to change.

*Community Care Treatment* Information was collected using an investigator-created form that captured the number of mental health appointments the Veteran had attended and the treatment modality.

*The Feasibility, Acceptability, and Satisfaction eScreening Survey*is a 15-item investigator-created survey comprised of six yes/no and nine Likert scale items designed to evaluate eScreening experience [16]. Items included questions about ease of use (e.g., length, text size, technical difficulties), acceptability, and satisfaction. See Table 3.

*Qualitative Interviews guides* were developed similar to those used in our other studies [13] to collect qualitative data on the feasibility and acceptability of eScreening as part of a measurement-based approach to care coordination of Veterans receiving mental health treatment in the community.

Veteran focus group interviews lasted approximately 90 min and included the following questions: “What have you liked about using eScreening?”; “What have you NOT liked about using eScreening?”; “Can you think of any improvements to eScreening that would make the experience better for you?”; “Have you found eScreening easy or difficult to use?”; “Did you have any technical difficulties using eScreening?”; “What do you think about having your eScreening results sent to your community provider?”; “Did you understand how your eScreening results would be used when you were invited into the project?”; and “Did you feel that the information that you're providing through eScreening has been redundant with what you also provide during treatment with your community care?”.

Individual interviews with CCP providers lasted approximately 30 min and included the following questions: “How would having regular symptom information be useful to you (or not)?”; “What would be the most helpful information to communicate?”; “What would be the most helpful way to communicate eScreening assessment results about your patients?”; “What or how much communication would you want from the VA Mental Health team?”; “Do you see potential drawbacks to this process?”; “Can you think of any improvements to this model?”.

### Data collection and analyses

This project used a pre-post mixed methods design to collect and analyze quantitative and qualitative data. Assessments followed the schedule in Table 1.

Quantitative data were characterized using descriptive statistics including means, standard deviations, frequencies, and percentages. Paired samples t-tests were used to examine changes in PCL-5, PHQ-9, and VR-12 scores between the initial assessment and follow-up assessments one and two, but not the final assessment which had a small number of respondents. All quantitative analyses were conducted using SPSS 27. Qualitative data from interviews were transcribed verbatim and coded by a project team member using a rapid qualitative analytic approach described by Hamilton and colleagues [19]. The data was used to describe and compare unique and common themes.

## Results

**Fig. 1:**
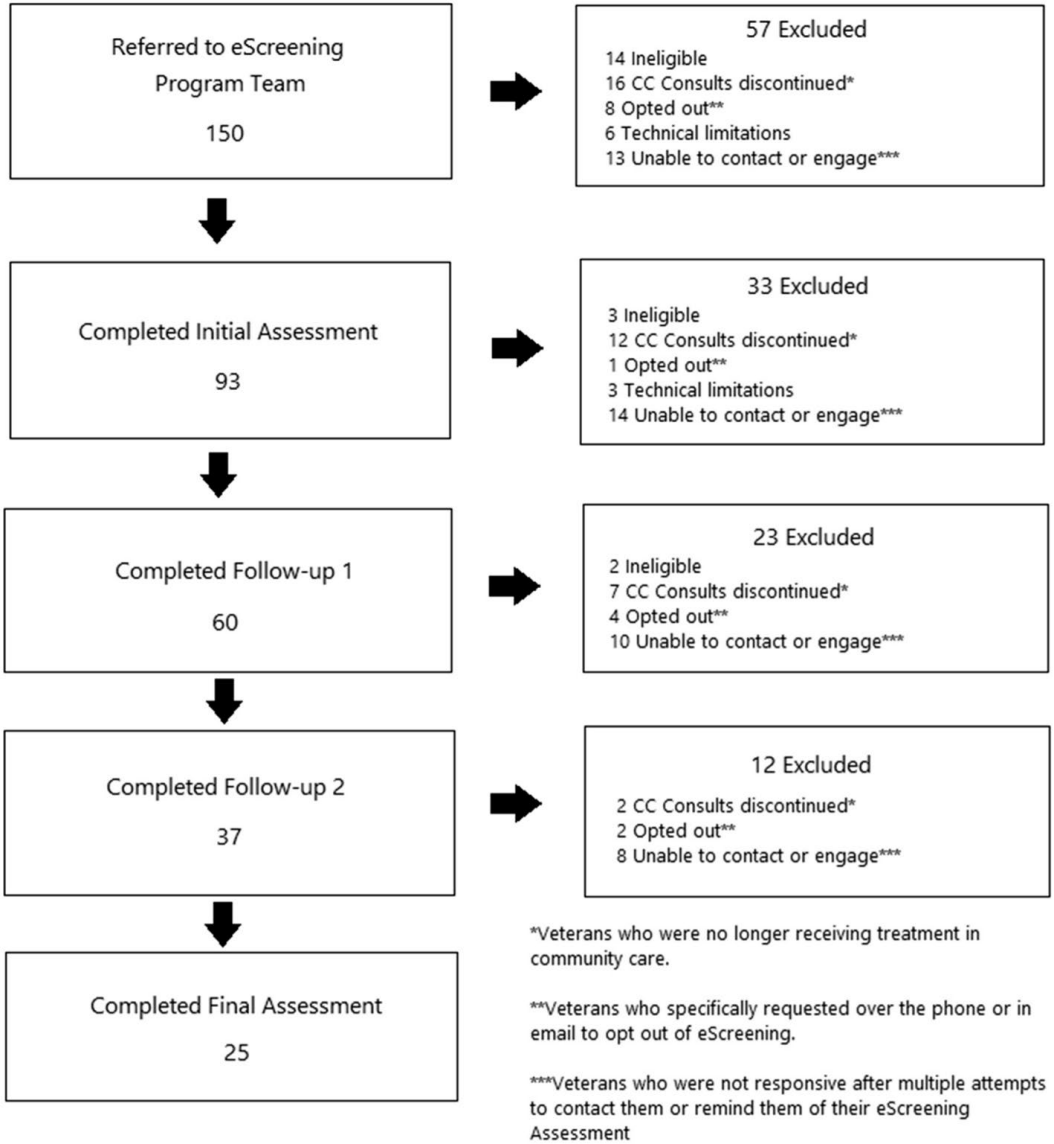
Participant flow diagram

A total of 150 Veterans were referred to participate in this project. Ninety-three (62%) Veterans completed the initial eScreening assessment, 60 (40%) completed the first follow-up assessment, 37 (24.7%) Veterans finished the second follow-up assessment, and 25 (16.7%) Veterans participated in the final assessment. The primary reasons for Veteran dropout at each timepoint was that they were either no longer participating in CCP, which resulted in the consult being discontinued, or unsuccessful attempts to contact Veterans. Figure 1 depicts assessment completers and non-completers.

### Demographics and mental health symptoms

The Veteran sample (*N* = 93) at the time of the initial assessment was 30% female. Regarding ethnicity and race, 27% of Veterans identified as Hispanic/Latino; 53% identified as White; 24% as Black/African American; 15% as Asian American; 6% as Pacific Islander/Native Hawaiian; 2% as American Indian; and 7% as other. Seventy-six percent of Veterans were somewhat to very comfortable answering questions about ethnicity and race using eScreening. Nine percent declined to answer. The PRO mental health data collected at each time point are summarized in Table 2 and includes only the Veterans who supplied complete data.

**Table 2:** Mental health measures by assessment time

Time Point	Measures	N	Mean	Std. Deviation
Initial Assessment	PCL-5^a^	56	44.12	19.00
	PHQ-9	86	13.95	6.44
	VR-12 PCS	80	21.51	3.46
	VR-12 MCS	81	21.74	3.00
Follow-up 1	PCL-5^a^	40	41.68	18.52
	PHQ-9	58	12.78	6.20
	VR-12 PCS	56	21.34	3.63
	VR-12 MCS	56	21.66	3.02
Follow-up 2	PCL-5^a^	26	40.73	17.35
	PHQ-9	37	12.27	5.64
	VR-12 PCS	37	20.81	3.33
	VR-12 MCS	37	21.00	2.29
Final Assessment	PCL-5^a^	16	34.81	20.99
	PHQ-9	22	11.95	5.48
	VR-12 PCS	21	21.76	3.74
	VR-12 MCS	20	21.65	2.76

^a^PCL5 was only administered to Veterans with a PTSD diagnosis. Ns are reflective of missing data

There were no significant differences in mental health symptom severity measures across assessment time points. However, there was a general trend toward improvement over time in PTSD and depression scores. This project led to 55 clinical encounters in which VA mental health staff called Veterans and conducted suicide screens (all negative) based on symptom scores. For each clinical encounter, VA mental health staff also called the Veteran’s CCP provider to communicate the clinically significant symptom increase. CCP providers did not have direct access to the PRO measures collected with eScreening. Instead, study staff reached out to CCP providers via phone call to share PRO data when it was clinically indicated. Study staff did not track if or how providers used the data as part of this project.

### Treatment history

Veterans were also asked questions about CCP. When asked how many sessions Veterans had with their VA CCP Mental Health provider at follow-up 1 (*N* = 60), 43% reported 0 sessions, 15% had 1 session, 12% had 2 sessions, 12% had 3 sessions, 8% had 4 sessions, 3% had 5 sessions, 2% had 6–7 sessions, 2% had 10 sessions, and 3% had 12 sessions. When asked to check all that apply, 35% participated in face-to-face sessions, 82% used video, 27% reported telephone, and 6% noted using some other format. By the final follow up assessment, all respondents (*N* = 25) reported at least one session with their VA CCP Mental Health provider; 13% had 2–3 sessions; 9% had 4 sessions; 13% had 5 sessions; 26% had 6 sessions; 9% had 8 sessions; and 4% had 10 and 12 sessions. Fifty seven percent reported face-to-face sessions; 73% used video; and 13% telephone.

### eScreening survey

Veterans also answered questions about their experience using the eScreening technology to complete PRO measures (Table 3 below). Of the Veterans who provided ratings at the first follow-up (Ns = 45 to 55), 89% had no technical difficulties; 78% felt comfortable entering personal information; 83% were neutral or positive about ease of use; and 89% were neutral or positive about the overall process.

**Table 3:** Veterans eScreening experience

Question	Disagree	Neither	Agree
eScreening meets my approval	17%	57%	26%
eScreening is appealing to me	29%	53%	18%
I like eScreening	28%	59%	13%
I welcome eScreening	24%	50%	26%
eScreening seems implementable	13%	40%	47%
eScreening seems possible	9%	38%	53%
eScreening seems doable	9%	38%	53%
eScreening seems easy to use	17%	34%	49%
**Question**	**Dissatisfied**	**Neither**	**Satisfied**
How satisfied are you with your overall eScreening experience?	11%	50%	39%
**Question**	**Yes**	**No**	
No issues, I successfully completed the eScreening evaluation	53%	47%	
The test took too long	11%	89%	
Technical difficulties with the website	11%	89%	
I did not feel comfortable entering personal information	22%	78%	
The text was too small and difficult to read	7%	93%	
Other	13%	87%	

### Qualitative interviews

#### Providers

Qualitative interview data were collected from seven CCP mental health providers. Email and electronic portals were among the preferred methods of communication, and providers were positive overall about eScreening. Providers indicated they found receiving symptom information from eScreening useful and noted it creates an opportunity for more communication, collaboration, and feedback. The providers stated the most helpful information to communicate is that pertaining to symptomology, benefit of and comfort with CCP treatment, communication from suicide prevention team/suicide risk flags, and anything to help “provide the best services for our clients”. Specific provider quotes included:

*It’s useful when patients can’t have regularly scheduled visits.*

*It's always useful to have symptom information.*

*It’s always useful to know where a Veteran is at emotionally, mentally, physically.*

*I think that you need to be collaborative, open to feedback, and continuously improving.*

*[We] might catch something new.*

*Well, it might be that you guys are catching something that I haven't caught, and that would be really important information, and I love that.*

*It would be nice if there were a case manager assigned to each person or someone that we can link up with to share information and…collaborate.*

#### Veterans

Focus group interview data were collected for 13 Veterans. Veterans liked knowing someone was monitoring their responses. They also liked the ease and convenience of using eScreening. Specific Veteran quotes included:

*One big thing is doing something and knowing that someone is looking into it. Because most of the time when I go to see my therapist, I have this feeling that I just completed these questionnaires, and no one is going to look at them.*

It's very easy. You can complete it at any time.

It's nice not to have to worry about missing a phone call.

You can save it, stop, and then continue later.

I would say convenience and the quickness of it.

I can sit at home and not be rushed.

No one's waiting on me and I can sit there and focus.

*You could just do it on your own, anytime, or anywhere. It's very accessible. You have time to think about your responses for all the questions, so there's no pressure. It's also very accommodating.*

*I believe it's more convenient. You can easily go online. You don't feel the sense of being probed by someone. You have the total control of everything, and you don't have this sense of being judged.*

One of the more frequent suggestions regarding eScreening was related to the questions and response options in the standardized measures and included statements such as:

*There's just middle of the road, good or excellent. You need something else in between because it's just not minutia enough.*

*Sometimes I get confused because the questions are kind of similar.*

Also, there was some concern regarding the communication of assessment results between VA and CCP providers:

*My biggest fear is the communication: how this can be transmitted to the community care provider themselves so they can see the proof of what they've been doing, if there is some improvement, or if there's something that they need to do. The communication is my biggest issue.*

## Discussion

Mental health conditions are common among Veterans and can have deleterious consequences across biopsychosocial domains including poorer physical health, homelessness, and suicidality [20–22]. It is imperative VA ensure Veterans receive quality treatment wherever they receive VA-related care. As VA endeavors to meet the demand for mental health services, a significant number of Veterans are accessing care through the VA CCP. Information on the quality of mental health care delivered to Veterans in CCP settings is scarce [18]. Health information technologies, such as eScreening, can support high quality care and Veteran safety in CCP settings by collecting, monitoring, and communicating PRO data. Thus, this project aimed to evaluate the feasibility and acceptability of using eScreening as part of a process involving remote symptom assessment, PRO monitoring, and clinically driven communication from VA to CCP providers, for Veterans accessing mental health treatment in CCP settings.

We successfully used eScreening to securely collect self-report demographic, mental health symptom (i.e., PCL-5, PHQ-9), functioning (VR-12), and satisfaction data from Veterans in CCP mental health settings. Survey feedback from Veterans about their eScreening experience was generally very positive. These findings are consistent with prior eScreening satisfaction research [10]. Qualitative data also were positive overall, and Veteran focus group data revealed that Veterans particularly liked the ease and convenience of using eScreening. Notably, a small percentage of Veterans had negative feedback which appeared to be primarily related to the content and format of the screening questions rather than the use of eScreening.

CCP mental health providers also supported the use of eScreening to collect symptom information and indicated that they are open to collaboration with VA. The collection of data via eScreening enables VA staff to monitor these data during a Veteran’s treatment in CCP and to communicate these data with CCP providers on a regular basis and when clinically significant changes (e.g., clinically significant increase in symptom or safety concerns) are reported by the Veteran. The eScreening program also uploads these data to the medical record system so that CCP treatment progress is available to all providers in the Veteran’s VA medical record. Given the versatility and increasing use nationally, eScreening is an ideal technology to remotely capture important self-report information from Veterans receiving CCP. The data can be integrated into the medical record and used to facilitate care coordination between Veterans, VA providers, and CCP providers. No other technology program has been used to facilitate collaborative care by remote capture of patient self-report data in CCP.

This project has several limitations. Due to low yield and Veteran feedback, we made adaptations to the recruitment and data collection procedures. Additionally, we did not know the reasons participants decided to not participate in CCP, which was a primary cause of attrition at each timepoint. The increase in satisfaction across timepoints could be a result of inflated scores by removal of potential dissenters to either CCP or eScreening, instead of an improvement in the appeal of the tool. Similarly, this self-electing bias for participating in qualitative interviews may skew feedback. In addition, this project did not track the number or amount of time study staff spent contacting Veterans for assessment reminders, answering questions, providing technical support, etc. Further, this project did not track clinical actions/outcomes related to the communication of PRO data. These will be important variables to include in future studies that use this model.

## Conclusion

With a significant portion of Veterans receiving care in the community, VA will require greater oversight, communication, and collaboration with treatment providers. Electronic screening is becoming a common practice throughout the private sector, and increasingly so within VA care. eScreening is a promising tool to maximize VA’s ability to enhance communication and potentially coordination of care with CCP providers. Future research is needed to evaluate the impact of using technology such as eScreening to facilitate measurement-based care coordination of Veterans accessing mental health treatment in the community. More research is also needed on increasing the transparency of VA’s communication with Veteran’s providers, developing measures which are acceptable to Veterans and clear in their purpose, and establishing acceptable schedules of assessment regarding frequency, redundancy, and actionable measurement. Nevertheless, technologies like eScreening represent a promising tool to support the mental health care Veterans receive when they access CCP.

## Data Availability

The datasets supporting the conclusions of this article are available from the corresponding author on reasonable request.

## Abbreviations

VA: Veteran Affairs
CCP: Community Care Program
MISSION: Maintaining Internal Systems and Strengthening Integrated Outside Networks
PRO: Patient reported outcomes
MBC: Measurement-based care
VASDHS: VA San Diego Healthcare System
QI: Quality improvement
PTSD: Post-traumatic stress disorder
MHSW: Mental Health Social Worker
PHQ-9: The Patient Health Questionnaire-9
PCL-5: The PTSD Checklist for DSM-5
VR-12: The Veterans RAND Health Survey
PCS: Physical Health Summary for the VR-12
MCS: Mental Health Summary Measure for the VR-12
SPSS: Statistical Package for Social Sciences
